# Histiocitosis congénita de células de Langerhans

**DOI:** 10.7705/biomedica.5150

**Published:** 2020-06-30

**Authors:** Katherine Barrios, Óscar Patiño, Nelson Muñoz, Carlos Moneriz

**Affiliations:** 1 Grupo de Investigación de Bioquímica y Enfermedad, Facultad de Medicina, Universidad de Cartagena, Cartagena, Colombia Universidad de Cartagena Facultad de Medicina Universidad de Cartagena Cartagena Colombia; 2 Hospital Infantil Napoleón Franco Pareja, Cartagena, Colombia Hospital Infantil Napoleón Franco Pareja Cartagena Colombia; 3 Departamento de Pediatría, Facultad de Medicina, Universidad de Cartagena, Cartagena, Colombia Universidad de Cartagena Facultad de Medicina Universidad de Cartagena Cartagena Colombia

**Keywords:** células de Langerhans, histiocitosis, recién nacido, lactante, Colombia, Langerhans cells, histiocytosis, infant, newborn, infant, Colombia

## Abstract

La histiocitosis de células de Langerhans es una enfermedad poco frecuente, cuyas manifestaciones clínicas pueden aparecer en el periodo neonatal y varían desde lesiones óseas aisladas hasta un compromiso sistémico.

Se describe un caso de histiocitosis de células de Langerhans y se revisa la literatura médica sobre las manifestaciones clínicas, el diagnóstico y el tratamiento. El paciente de un mes de nacido fue llevado a consulta por presentar adenopatías y lesiones en la piel que, inicialmente, fueron tratadas como reacción a una infección. La enfermedad continuó su progresión sin que hubiera mejoría con el tratamiento, hasta que el paciente falleció por falla respiratoria.

La biopsia de ganglio linfático y la de piel revelaron infiltración de células atípicas, y la inmunohistoquímica resultó positiva para las proteínas S1OO, CD1 y CD68, con lo cual se confirmó el diagnóstico de histiocitosis de células de Langerhans.

Esta alteración representa un gran desafío clínico, por lo que es importante alertar y sensibilizar al equipo médico para lograr un diagnóstico y un tratamiento más oportunos.

## Abstract (en)

Langerhans cell histiocytosis is a rare pathology with different clinical manifestations in the neonatal period ranging from isolated bone lesions to systemic compromise.

We report a case of Langerhans cell histiocytosis including a literature review focused on the clinical manifestations, diagnosis, and treatment. A one-month-old patient was brought to medical consultation with lymphadenopathy and skin lesions, which were initially managed as an infectious pathology. The disease continued its progression without improvement with the treatment until the patient died due to respiratory failure.

The lymph node and skin biopsies revealed infiltration of atypical cells with positive immunohistochemistry for S1OO, CD1, and CD68 confirming Langerhans cell histiocytosis. This disorder represents a great challenge and, therefore, it is important to alert and sensitize medical teams about it for timely diagnosis and management.

La histiocitosis de células de Langerhans es un trastorno poco frecuente del sistema monocito-macrófago caracterizado por la proliferación descontrolada de una estirpe de células dendríticas inmaduras positivas para CD1a/CD2O7 en piel, huesos, ganglios linfáticos y otros órganos ^1^^,^^2^. Se la denomina así por la morfología de las células patológicas, las cuales se asemejan a las células de Langerhans, aunque originadas en células mieloides ^3^.

Su etiología sigue siendo desconocida y la hipótesis más aceptada es la desregulación del sistema inmunitario y la transformación maligna de las células dendríticas por mecanismos de mutación genética ^3^^,^^4^. Las lesiones que produce contienen células de Langerhans, así como células inflamatorias como linfocitos T, macrófagos, células plasmáticas, eosinófilos, células gigantes multinucleadas similares a osteoclastos y neutrófilos ^5^^,^^6^ (figura 1).

**Figura 1:**
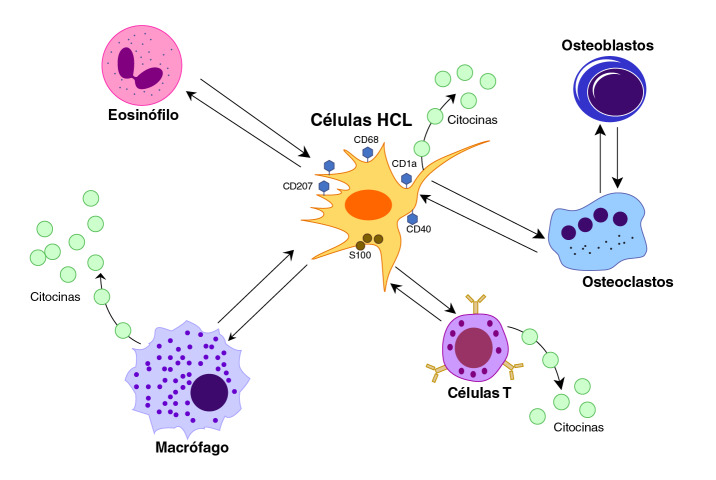
Interacción celular en la histiocitosis de células de Langerhans. Las lesiones se caracterizan por una liberación descontrolada de citocinas y quimiocinas e interacciones con células infiltradas, las cuales desempeñan un papel en la acumulación de células de Langerhans y son las responsables de las manifestaciones clínicas de la enfermedad. Las células de Langerhans en la histiocitosis expresan principalmente las proteínas CD1a, CD40, CD68, CD207 y S100.

La enfermedad se caracteriza por una variedad de manifestaciones, únicas o múltiples, con infiltración de histiocitos capaces de afectar casi todos los órganos, especialmente piel, ganglios linfáticos, pulmones, timo, hígado, bazo, médula ósea y sistema nervioso central ^7^^,^^8^, por lo que es necesario integrar la clínica, los estudios histopatológicos y la inmunohistoquímica para su detección oportuna ^9^.

Se reporta el caso de un niño lactante a quien se le diagnosticó histiocitosis de células de Langerhans después de su fallecimiento por una complicación respiratoria de la enfermedad. El objetivo de informar este caso es alertar a los equipos médicos sobre la necesidad de conocer mejor la enfermedad y contemplarla como una posibilidad diagnóstica, con el fin de ofrecer un tratamiento oportuno y disminuir la morbilidad y la mortalidad en estos pacientes.

## Caso clínico

**Figura 2:**
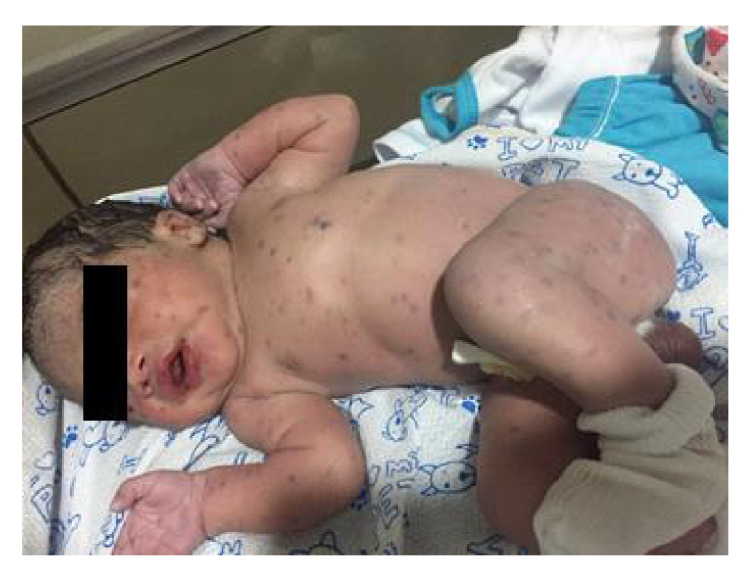
Histiocitosis de células de Langerhans del paciente a los pocos días de nacido. Lesiones en toda la superficie cutánea en forma continua, principalmente en cara, abdomen, brazos y piernas, con características papulares violáceas, aproximadamente, de 0,5 cm de diámetro y sin afectación de las mucosas

Se presenta el caso de un lactante masculino de un mes y diez días de edad, procedente de una zona del sur de Bolívar (Colombia) y remitido de la consulta externa del Hospital Infantil Napoleón Franco Pareja de Cartagena por presentar adenopatías generalizadas de características inflamatorias. Entre los antecedentes, los familiares informaron que el niño había estado dos semanas en la unidad de cuidados intensivos después de su nacimiento y allí presentó pápulas violáceas generalizadas en la piel (figura 2). En las pruebas para el perfil TORCH hechas en otra institución de salud, la madre había sido positiva para IgG, pero negativa para IgM y no había habido otros hallazgos analíticos, serológicos o ecográficos significativos.

Después de una hospitalización del paciente durante de 26 días, se sospechó infección congénita por citomegalovirus, se le administró el tratamiento y se le dio de alta con controles por infectología.

**Figura 3:**
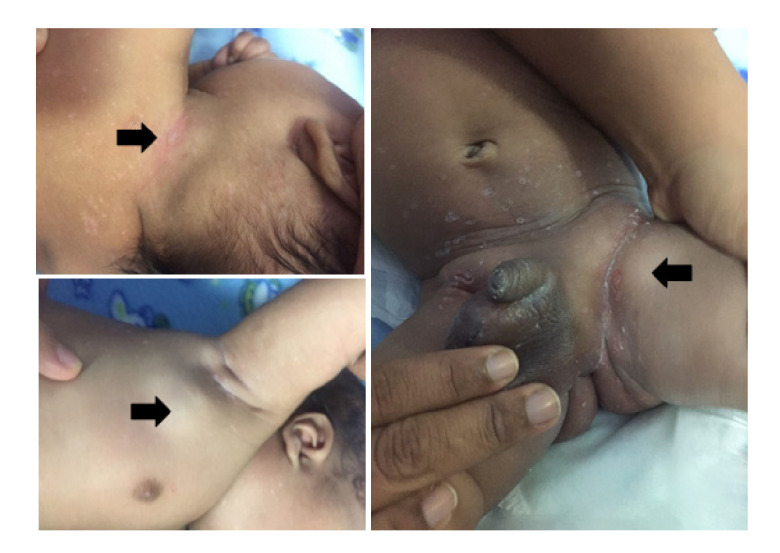
Histiocitosis de células de Langerhans del paciente al mes de vida. Lesiones hipocrómicas en toda la superficie corporal y adenopatías generalizadas en las regiones cervical anterior, axilar e inguinal. Las flechas muestran adenopatías de aproximadamente 2,5 cm en la región cervical posterior, de 1 a 2 cm en la región axilar izquierda y del mismo tamaño en las dos zonas inguinales. Se observan en la piel lesiones generalizadas vesículo-ampollosas del tipo de pápulas descamativas.

Al mes y diez días de edad, el niño fue llevado a consulta externa en el Servicio de Infectología y allí se evidenció la progresión de la enfermedad con lesiones hipocrómicas en toda la superficie corporal y adenopatías generalizadas (figura 3). El paciente ingresó en aceptables condiciones generales: tenía estabilidad hemodinámica, estaba afebril, no presentaba signos de dificultad respiratoria, toleraba la vía oral y no había signos activos de síndrome de reacción inflamatoria sistémica ni presencia de úlceras bucales.

Al persistir la sospecha clínica de infección, se hospitalizó, se le dio lactancia materna exclusiva y se hizo el seguimiento multidisciplinario. A las 48 horas de la hospitalización, en el hemograma se evidenció anemia normocítica y normocrómica, pero el resto de los exámenes paraclínicos y otras pruebas estuvieron dentro de límites normales. Se hizo la prueba de serología para el perfil TORCH, con resultados de IgG positiva para toxoplasma y citomegalovirus, aunque la carga viral para este último arrojó un resultado negativo. En la ecografía de tejidos blandos se apreció la presencia de adenopatías y se ordenó una biopsia de ganglio y de piel por sospecha de síndrome linfoproliferativo. Seis días después de la hospitalización, el paciente presentó picos febriles, tos y congestión nasal. En la auscultación se encontraron leves sibilancias en las dos bases pulmonares, por lo que se le administró salbutamol.

En esta etapa, la biopsia del ganglio axilar izquierdo reveló proliferación de células linfoides atípicas con núcleos grandes y vacuolados y patrón difuso, con extensión y compromiso del seno subcapsular, así como un índice mitótico elevado y mitosis atípicas. En la biopsia de piel, se observó textura tapizada debida al epitelio escamoso con infiltración de células mononucleares atípicas dispuestas en nidos en diferentes niveles.

**Figura 4:**
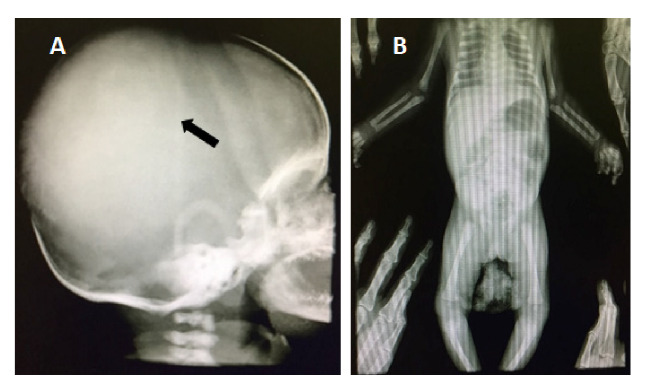
Radiografía de cráneo y tórax del paciente con histiocitosis de células de Langerhans. **A.** Radiografía de cráneo que denota aumento del volumen de los huesos de la bóveda craneana y desproporción craneofacial. **B.** Radiografías normales de tórax y huesos largos

El Servicio de Hematooncología descartó el diagnóstico de linfoma dado que no es característico en el rango de edad del paciente, y los síntomas y el curso clínico concordaban más con histiocitosis, por lo cual se hicieron pruebas de inmunohistoquímica y se obtuvieron radiografías de tórax, cráneo y huesos largos, en las cuales se observó incremento del volumen de los huesos de la bóveda craneana y la desproporción craneofacial (figura 4). Dado el resultado de patología, se inició la quimioterapia prefase con prednisona y ciclofosfamida.

Durante el tratamiento el niño presentó sibilancias asociadas con dificultad respiratoria, taquipnea, tirajes y adenopatías cervicales grandes con riesgo de obstrucción de la vía aérea superior, por lo que se le trasladó a la unidad de cuidados intensivos. Allí se tornó tórpido y presentó neutropenia febril grave y bacteriemia por *Staphylococcus aureus*, lo que desembocó en una bradicardia extrema sin respuesta a las maniobras de reanimación cardiopulmonar y, finalmente, en la muerte del niño tras cinco días en la unidad de cuidados intensivos.

Pocos días después de la defunción, el estudio hepatológico reveló tejido ganglionar con un infiltrado constituido por histiocitos de núcleo grande, hipercromático, acompañado de neutrófilos y eosinófilos. En el microscopio electrónico se observaron histiocitos de núcleo irregular y citoplasma amplio y rico en lisosomas acompañados de cuerpos bastoniformes circunscritos por membrana, en cuyo seno aparecían con periodicidad espacial ciertas granulaciones. El análisis por inmunohistoquímica de estas células mostró su reacción a las proteínas S100, CD1 y CD68, con lo cual se confirmó el diagnóstico de histiocitosis de células de Langerhans.

### Consideraciones éticas

Los autores manifiestan que los procedimientos seguidos se ajustaron a las normas éticas del comité de experimentación humana responsable, a las de la Asociación Médica Mundial y a las disposiciones de la Declaración de Helsinki. Se siguieron los protocolos del centro de trabajo de los autores para la publicación de los datos de los pacientes y se obtuvo el consentimiento informado de los cuidadores del paciente.

## Discusión

La histiocitosis de células de Langerhans es un trastorno raro cuya incidencia real se desconoce ^7^, aunque se reporta una de 3 a 5 casos por millón, especialmente en la infancia. En el periodo neonatal, esta es, aproximadamente, de 1 a 2 casos por millón ^10^ y es ligeramente superior en hombres ^4^. La incidencia real en Colombia no se conoce.

La presentación clínica de la enfermedad varía dependiendo del sitio y el grado de afectación. La enfermedad se manifiesta en un solo sistema de órganos, aproximadamente, en el 55 % de los pacientes, en tanto que en el porcentaje restante se presenta como una enfermedad multisistémica ^11^^-^^13^; es más común en menores de tres años. Su manifestación en un único órgano es más común en niños mayores y en adultos ^13^.

En los niños, la histiocitosis de células de Langerhans puede afectar cualquier hueso del cuerpo, sobre todo el cráneo (40 %), el fémur**,** las costillas, las vértebras y el húmero ^11^. En el caso en estudio, hubo aumento de volumen de los huesos de la bóveda craneana y desproporción craneofacial, pero no hubo afectación de huesos largos u otra estructura ósea.

Aproximadamente, en el 20 % de los pacientes se presenta linfadenopatía que involucra principalmente los ganglios cervicales ^13^, lo que fue evidente en el presente caso por las adenopatías generalizadas.

Las manifestaciones cutáneas más comunes son pápulas marrones, púrpura y erupción eccematosa semejante a la de una infección por cándida. Otras lesiones cutáneas incluyen pústulas purpúricas, petequiales, vesiculares o pápulo-nodulares ^14^, que se pueden presentar en el 60 % de los casos ^4^ y que también se presentaron en este paciente (figuras 2 y 3).

La incidencia de afectación de la médula ósea en pacientes con esta condición es de alrededor del 34 %. La mayoría de los pacientes con afectación de la médula ósea son niños pequeños con enfermedad difusa en el hígado, el bazo, los ganglios linfáticos y la piel ^11^. Dicha manifestación puede darse como neutropenia, anemia y trombocitopenia en el 15 al 30 % de los casos ^4^. En el presente caso, se determinó compromiso de la medula ósea por la presencia de neutropenia, lo que complicó el estado clínico del paciente al detectarse bacteriemia por *S. aureus.*

La afectación del sistema respiratorio es relativamente infrecuente, apenas en el 15 % de los casos, pero es una de las principales causas de mortalidad en estos pacientes ^4^. En el curso clínico del paciente, se presentó un cuadro clínico de sibilancias, taquipnea y tirajes que evolucionó a un síndrome de dificultad respiratoria, lo que con la bacteriemia por *S. aureus,* indujo una respuesta inflamatoria sistémica con bradicardia extrema sin respuesta a las maniobras de reanimación y, finalmente, la muerte del paciente.

Otras manifestaciones clínicas descritas en la histiocitosis de células de Langerhans, las cuales no se presentaron en el presente caso, son por compromiso del bazo, con esplenomegalia en el 15 % de los casos, del hígado, con hepatomegalia y falla hepática en el 15 % de los casos, del sistema hipotálamo-hipofisiario, con diabetes insípida, hidrocefalia y parálisis de pares craneales, entre otros, en alrededor del 25 % de los casos, y, por último, del sistema digestivo, con diarrea hemorrágica en menos del 5 % de los pacientes ^4^.

El diagnóstico se confirma mediante los estudios histopatológicos y el análisis por inmunohistoquímica con resultado positivo para las moléculas CD1 a CD207 (langerina) características de las líneas dendríticas, así como mediante otros marcadores de la línea mieloide, como las proteínas S100 y CD68 ^9^^,^^10^^,^^15^. La historia clínica, el examen físico y las pruebas de laboratorio deben correlacionarse para clasificar al paciente según el grado de afectación y, así, proceder con el tratamiento ^5^.

En el caso que se presenta, y dada la sospecha clínica de histiocitosis de células de Langerhans a partir del estudio de hematooncología, la enfermedad se clasificó como multisistémica por afectar en ese momento la piel y el sistema óseo, aunque se la consideró de bajo riesgo al no haberse detectado la afectación de un órgano de riesgo. Actualmente, para la clasificación de la enfermedad como sistémica, se recomiendan los estudios de tomografía que, en este caso, no fue posible realizar. Al progresar, la enfermedad se consideró multisistémica de alto riesgo, pues afectó los pulmones y el sistema hematopoyético con un síndrome de dificultad respiratoria, anemia y neutropenia.

La progresión continua de las lesiones cutáneas en esta enfermedad no necesariamente indica gravedad, pero se recomienda hacer seguimiento de los pacientes, inclusive después de que hayan desaparecido. La condición tiene un curso clínico impredecible dada la ausencia de factores predictores clínicos y de laboratorio que detecten a los pacientes con riesgo de desarrollar la variación multisistémica y sus posibles complicaciones ^4^.

Para el tratamiento se tiene en cuenta la clasificación de riesgo. Cuando la enfermedad es de un único sistema, las opciones de tratamiento incluyen prednisona como único agente, la combinación de vinblastina y prednisona, legrado de las lesiones óseas y tratamiento tópico de las lesiones cutáneas. Si la enfermedad es multisistémica, se administra una fase inicial de quimioterapia con vinblastina y prednisona durante seis semanas ^16^. Los pacientes que mejoren adecuadamente continúan con un ciclo de 12 meses de vinblastina y prednisona, y se añade mercaptopurina en aquellos con compromiso de un órgano en riesgo ^17^.

En el presente caso se administró la fase inicial de quimioterapia con prednisona y ciclofosfamida, que comúnmente sustituye la vinblastina por ser un regulador del sistema inmunitario con características de agente citostático alquilante útil en el control de la proliferación de las células mieloides ^18^. En este paciente, se decidió utilizar un tratamiento de rescate debido a la gravedad de su cuadro clínico y la poca respuesta terapéutica. En este tipo de terapia, se podrían usar distintas combinaciones de dexorrubicina, ciclofosfamida, metotrexato, vincristina y prednisolona.

En estudios previos se ha evidenciado que, con esta estrategia terapéutica se logra una tasa de recuperación de la enfermedad multisistémica del 78 % a los cinco años y un promedio de supervivencia del 95 %, con una disminución significativa de la mortalidad ^19^^,^^20^, lo que podría atribuirse al cambio temprano a un tratamiento de rescate más efectivo en pacientes con poca mejoría, así como a un mejor respaldo clínico. En otros estudios, se ha determinado una mortalidad de cerca del 35 % en pacientes con la enfermedad multisistémica de alto riesgo (compromiso de hígado, bazo o médula ósea), pese a la administración del tratamiento correcto durante las primeras seis semanas, aunque se ha encontrado una supervivencia promedio del 84 % en pacientes tratados por 12 meses con quimioterapia ^21^^,^^22^.

Pese a estas recomendaciones de tratamiento, no existe consenso sobre cuál es el óptimo, especialmente cuando la enfermedad es multisistémica. Debido a las diversas presentaciones, la *Histiocyte Society* recomienda que el tratamiento debe adaptarse a las características de cada presentación y a la respuesta al tratamiento en los diferentes estadios de la enfermedad.

En conclusión, la histiocitosis de células de Langerhans es una enfermedad rara de las células mieloides que puede afectar a cualquier grupo de edad y abarca un amplio espectro de manifestaciones locales y sistémicas, dependiendo del estadio y los órganos comprometidos. Su causa es desconocida y se caracteriza por proliferación de las células de Langerhans. Tiene diferentes manifestaciones radiológicas y, muchas veces, el primer estudio diagnóstico corresponde a la radiografía simple de la zona afectada, cuyos hallazgos pueden ser difíciles de interpretar y requieren confirmación mediante los análisis histopatológicos. Por ello, la enfermedad representa un desafío y es importante sensibilizar al personal médico sobre la necesidad de profundizar en su conocimiento para un diagnóstico y manejo oportunos.
